# Healing of a bone-exposed soft tissue defect after surgical correction of traumatic patellar dislocation: A case report

**DOI:** 10.1097/MD.0000000000036324

**Published:** 2023-11-24

**Authors:** Kuan-I Lee, Yun-Nan Lin, Yu-Chuan Lin, Yur-Ren Kuo

**Affiliations:** a School of Medicine, Kaohsiung Medical University, Kaohsiung, Taiwan; b School of Post-Baccalaureate Medicine, College of Medicine, Kaohsiung Medical University, Kaohsiung City, Taiwan; c Division of Plastic Surgery, Department of Surgery, Kaohsiung Medical University Hospital, Kaohsiung Medical University, Kaohsiung, Taiwan; d Department of Orthopedic Surgery, Kaohsiung Medical University Hospital, Kaohsiung Medical University, Kaohsiung, Taiwan.

**Keywords:** bone exposure, collagen dressings, lower extremity, patellar dislocation, soft tissue defects

## Abstract

**Rationale:**

Reconstructive surgery is widely considered the primary treatment for soft tissue defects around the knee owing to its high flexibility. However, in our recent case study, we explored an alternative approach using decellularized collagen dressings, which proved highly effective in healing a soft tissue defect involving bone exposure following surgical correction of a traumatic patellar dislocation.

**Patient concerns:**

A 65-year-old male with a traumatic patellar dislocation in the lower extremity failed to approximate the wound after surgical correction. The patient refused additional surgical reconstruction because of the potential risks of multiple operative complications.

**Diagnoses:**

Traumatic patellar dislocation complicated by exposed bone following surgical treatment was made.

**Interventions:**

The procedure was performed using ABCcolla® Collagen Matrix (ACRO Biomedical, Taiwan), an acellular dermal matrix made from a decellularized native porcine collagen scaffold dressing. Collagen dressings were applied to the soft tissue defect, and biointegration was observed in the wound area of bone exposure.

**Outcomes:**

Through the application of ABCcolla® Collagen Matrix (ACRO Biomedical, Taiwan) and diligent wound care for a total of 105 days, the patient healed successfully and achieved partial functional recovery after undergoing rehabilitation. During recent outpatient clinic visits, the patient is now able to ambulate independently with the aid of crutches.

**Lessons:**

Collagen dressings circumvent the potential risks and complications associated with multiple surgical procedures. We believe that the utilization of collagen dressings, combined with careful wound management, could serve as a promising alternative treatment option for patients with soft tissue defects around the knee in the future.

## 1. Introduction

Patellar dislocation accounts for approximately 3% of all knee injuries.^[1]^ Young, active individuals, particularly females, are at a higher risk of patellar dislocation.^[1]^ Lateral patellar dislocation is the most common type of patellar dislocation due to its anatomical structures and is typically associated with a rupture of the medial patellofemoral ligament and medial patellar retinaculum.^[2]^ The reported incidence of first-time lateral patellar dislocation is 23 per 100,000 person-years,^[3]^ with almost half of all dislocations being caused by sports and accident-related trauma.^[4]^ Notably, more than one-third of patients with first-time lateral patellar dislocation experience recurrence within a few years.^[5]^

Closed reduction and immobilization are typically recommended for patients with patellar dislocations without osteochondral fractures.^[6]^ If closed reduction fails, open reduction and repair of soft tissue structures are necessary.^[7,8]^ Reconstructive surgeries are mandatory to address soft tissue defects following correction around the knee.^[9–11]^

In this study, we presented the case of a 65-year-old male who developed a significant soft tissue defect after undergoing surgical correction for a recurrent left lateral patellar dislocation. The injury occurred when the patient was severely hit by a propeller while diving. We demonstrated a novel approach using decellularized collagen dressings to achieve successful wound healing in this challenging-to-close wound.

## 2. Case report

The patient was a 65-year-old male with a medical history of hypertension, dyslipidemia, and myocardial infarction. The patient was transferred to the emergency department after being hit by a boat-propeller while diving. Upon arrival, the patient exhibited massive bleeding, multiple fractures, and left knee dislocation with malalignment. Imaging studies revealed bilateral femoral shaft fractures, pelvic fractures, and a contusion in the left knee with ligament rupture. The orthopedic surgeon performed an open reduction of the femoral and pelvic fractures. Following the operation and 1 week of postoperative care, the patient was discharged from the hospital and attended regular follow-up visits at the orthopedic outpatient clinic.

**Figure 1.**
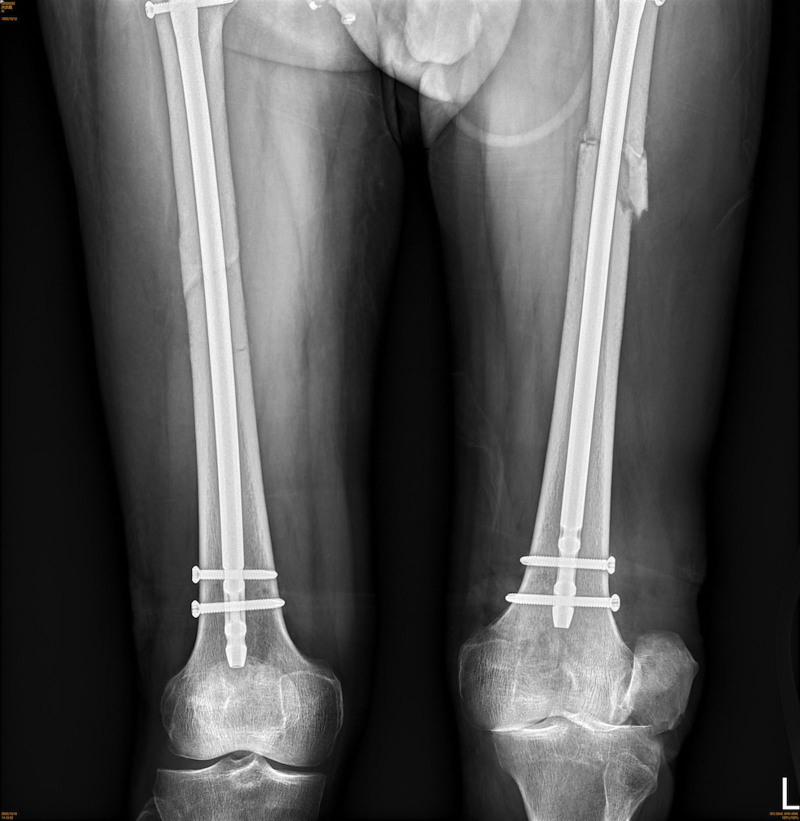
The X-ray image shows patellar dislocation following open reduction of the pelvic and bilateral femoral fractures caused by boat-propellers.

During the follow-up visits, the patient reported a limited range of motion in the left knee joint, tenderness, and swelling. A diagnosis of a left patellar dislocation was made (Fig. 1). An attempt at a closed reduction of the left patella was unsuccessful. Subsequently, an open reduction and capsular release of the patella was performed, but the patella remained dislocated. To correct the position of the patella, the orthopedic surgeon performed the same procedure a second time, along with a medial patellofemoral ligament augmentation. However, the approximation of the skin edges was failed.

**Figure 2.**
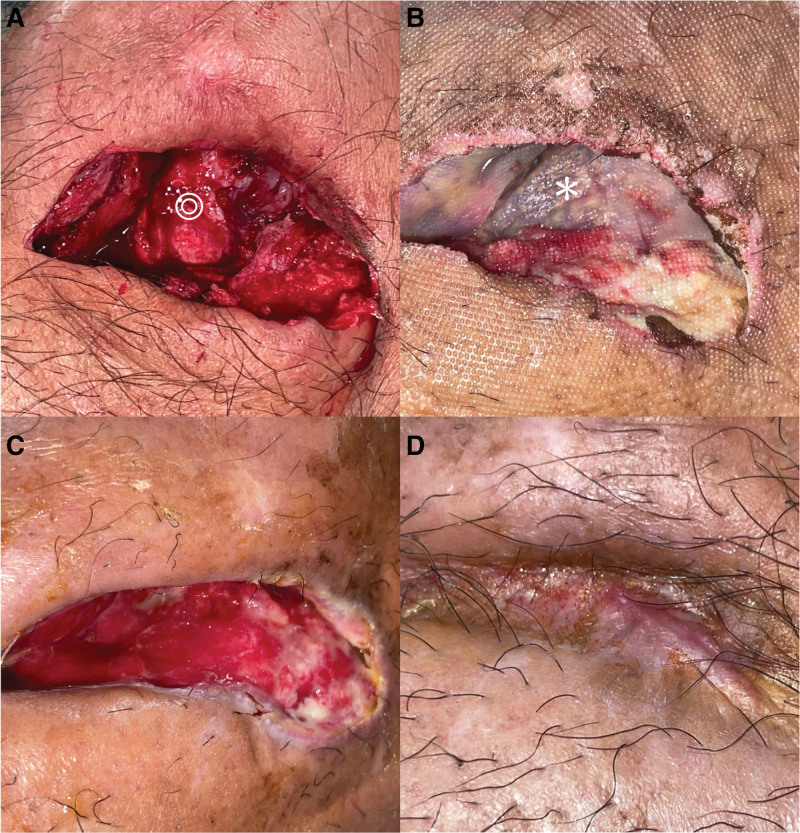
Healing process of a challenging soft tissue defect after surgical correction of patellar dislocation. (A) The soft tissue defect wound with exposed bone. (B) Application of acellular dermal matrix to the wound. (C) Progress of biointegration in the bone-exposed area. (D) The wound attained full healing following a 105-day treatment regimen, which involved the use of acellular dermal matrix and careful wound management. (◎ bone;* acellular dermal matrix).

**Figure 3.**
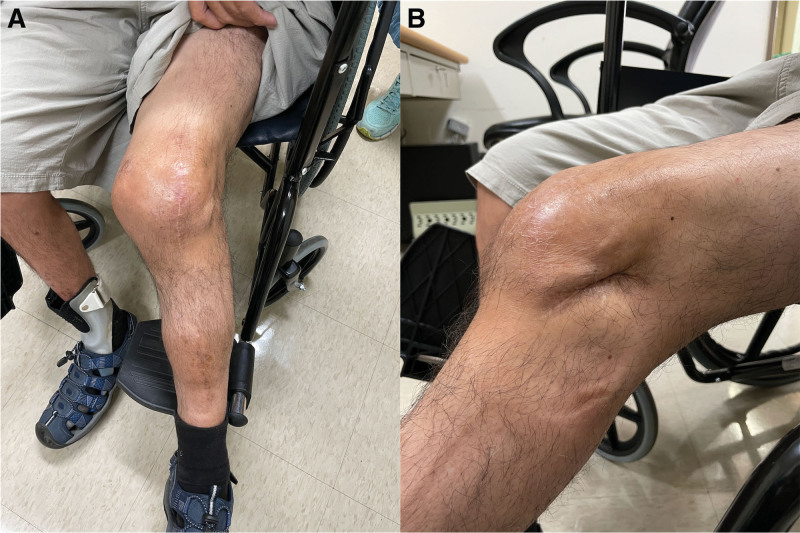
Patient was satisfied with the appearance of the scar during 2-year follow-up visits at the outpatient department clinic. (A: anterior view; B: lateral view).

Considering the patient’s previous surgeries and the uncertainty regarding the success of intensive reconstructive surgeries, the patient declined flap reconstruction. In this case, we utilized an acellular dermal matrix (ADM), specifically the decellularized collagen dressings called ABCcolla® Collagen Matrix (ACRO Biomedical, Taiwan) daily, for the treatment of the exposed bone wounds, which measured 9.1 cm^2^ before treatment. After 2 months of treatment, the wound size gradually decreased to 2.9 cm^2^. After 105 days of meticulous wound management, the wound finally healed successfully (Fig. 2) and no adverse events and other unanticipated problems were observed. During the patient’s most recent follow-up, it was observed that the left knee had a range of motion of 0 to 75 degrees. The patient underwent rehabilitation and was able to walk with the assistance of crutches (Movie S1, Supplemental Digital Content, http://links.lww.com/MD/K902). The patient expressed satisfaction with the postoperative scar appearance and functional outcome (Fig. 3). Written informed consent was obtained from the patient for publication of this case report and accompanying photos.

## 3. Discussion

This study presents the case of a patient who survived a boat-propeller accident and developed a challenging soft tissue defect that was difficult to close following surgical correction. The patient achieved successful healing using decellularized collagen dressings. Although boat propeller injuries are rare, they can have severe consequences, including deep lacerations, extensive scarring, significant blood loss, and even amputations. These injuries can even be fatal.^[12]^ The overall mortality rate for propeller injuries ranges from 15% to 17%, with similar rates for major amputations.^[13]^ Although the patient survived amputations, poor functional recovery from propeller-induced injuries was predictable.^[14]^

Survivors of boat-propeller related injuries typically require a series of complex reconstructive surgeries, extended hospital stays, and extensive rehabilitation to regain functionality. However, one of the challenges that arise after surgery is tissue swelling and the development of soft tissue fibrosis due to multiple surgical interventions. These factors impede the approximation and closure of wounds.

Soft tissue defects around the knee often arise from significant trauma, extensive tumor excision, or postburn contractures. It is important to note that reconstructing the area around the patella presents a unique challenge. The focus is not only on addressing the defect itself but also on ensuring adequate knee joint flexibility for normal walking and mobility.^[15,16]^ Although muscle coverage and flap reconstruction are considered standard procedures, they are associated with risks such as flap necrosis, donor site complications, and other potential complications.^[17,18]^

The ABCcolla® Collagen Matrix is a type of ADM made from decellularized native porcine collagen scaffold dressing that has been reported to revolutionize the wound healing process.^[19]^ The biochemical functions of ADM are attributed to its extracellular matrix.^[20]^ Type I collagen, a major component of the extracellular matrix, plays a crucial role in wound healing by modulating cellular functions.^[21]^ Previous studies have shown that type I collagen can protect wounds from deterioration by binding to free radicals, proteases, and inflammatory cytokines in the wound bed.^[22]^ It has been reported to protect vital structures, reduce wound closure time, and stimulate the growth of new functional dermis.^[23]^ ADMs have been widely used to reconstruct soft tissue defects in various contexts, such as diabetic ulcers, pressure ulcers, chest walls, abdominal walls, and even maxillofacial surgery.^[23,24]^ However, the applications to the knee have rarely been mentioned.

To the best of our knowledge, this is the first reported case in which decellularized collagen dressings were used to heal a soft tissue defect around the knee, a joint known for its high flexibility, after surgical correction. The remarkable outcome of this case not only achieved an aesthetically pleasing result but also led to an acceptable level of functional recovery after a rehabilitation program. The successful application of decellularized collagen dressings highlights their potential as a valuable treatment option for similar cases, demonstrating their ability to promote tissue regeneration and restore both appearance and function. Further research and clinical studies are needed to validate these findings and to explore the broader implications and benefits of decellularized collagen dressings in the management of soft tissue defects around the knee.

It is important to acknowledge the limitations of this study. Firstly, the technique employed may not be applicable to all soft tissue defects, especially those of significant size. There are inherent limitations to using decellularized collagen dressings to heal large defects, and alternative approaches may be necessary in such cases. In addition, our study represents a single case, and while the results are promising, it is crucial to conduct additional case–control studies with a larger number of patients. Robust research with a large sample size will allow for more accurate conclusions and assessment of the reliability and reproducibility of this technique.

Our study highlights the potential of collagen dressings as an alternative treatment option for patients with flexible areas affected by bone-exposed soft tissue defects. This finding emphasizes the possibility of successful healing and achieving partial functional recovery without the need for surgical correction. Although surgical intervention is frequently necessary, our findings suggest that diligent wound care and the use of collagen dressings can effectively promote healing in these cases.

## 4. Conclusion

In summary, our study adds to the existing body of evidence supporting the potential use of collagen dressings as an alternative treatment modality for bone-exposed soft tissue defects. These findings provide optimism for improved outcomes and enhanced future patient care.

## Acknowledgments

We would like to thank the patient who participated in this study.

## Author contributions

**Conceptualization:** Yun-Nan Lin.

**Resources:** Yun-Nan Lin, Yu-Chuan Lin.

**Supervision:** Yun-Nan Lin, Yur-Ren Kuo.

**Visualization:** Yun-Nan Lin, Yur-Ren Kuo.

**Writing – original draft:** Kuan-I Lee.

**Writing – review & editing:** Kuan-I Lee, Yun-Nan Lin, Yu-Chuan Lin, Yur-Ren Kuo.

## Supplementary Material
